# The role of physical and mental multimorbidity in suicidal thoughts and behaviours in a Scottish population cohort study

**DOI:** 10.1186/s12888-019-2032-8

**Published:** 2019-01-23

**Authors:** Katerina Kavalidouª, Daniel J. Smith, Geoff Der, Rory C. O’Connor

**Affiliations:** 1 Suicidal Behaviour Research Laboratory, Institute of Health & Wellbeing, University of Glasgow, Glasgow, UK; 2 Institute of Health & Wellbeing, University of Glasgow, Glasgow, UK; 3 MRC/CSO Social & Public Health Sciences Unit, University of Glasgow, Glasgow, UK; 4 Hull York Medical School, University of Hull, Hull, HU6 7RX UK

**Keywords:** Suicidal thoughts, Suicide attempts, Multimorbidity, Health conditions, Cohort

## Abstract

**Background:**

Physical illness and mental disorders play a significant role in fatal and non-fatal suicidal behaviour. However, there is no clear evidence for the effect of physical and mental illness co-occurrence (multimorbidity) in suicidal ideation and attempts. The aim of the current study was to investigate, whether physical/mental health multimorbidity predicted suicidal thoughts and behaviours as outcomes.

**Methods:**

Data from the West of Scotland Twenty-07 cohort were analysed. Twenty-07 is a multiple cohort study following people for 20 years, through five waves of data collection. Participants who responded to past-year suicidal thoughts and suicide attempt items were grouped into four distinct health-groups based on having: (1) neither physical nor mental health condition (controls); (2) one or more physical health condition; (3) one or more mental health condition and; (4) multimorbidity. The role of multimorbidity in predicting suicidal ideation and suicide attempts was tested with a generalised estimating equation (GEE) model and odds ratios (ORs) and 95% CIs are presented. Whether the effect of multimorbidity was stronger than either health condition alone was also assessed.

**Results:**

Multimorbidity had a significant effect on suicidal thoughts and suicide attempts, compared to the control group, but was not found to increase the risk of either suicide-related outcomes, more than mental illness alone.

**Conclusions:**

We identified an effect of physical/mental multimorbidity on risk of suicidal thoughts and suicide attempts. Considering that suicide and related behaviour are rare events, future studies should employ a prospective design on the role of multimorbidity in suicidality, employing larger datasets.

**Electronic supplementary material:**

The online version of this article (10.1186/s12888-019-2032-8) contains supplementary material, which is available to authorized users.

## Background

Suicide is a complex phenomenon, involving the interaction between genetic, neurobiological, psychological, behavioural and environmental risk/protective factors [1–4]. Epidemiological research has indicated that risk of suicidal behaviour and suicide varies as a function of sociodemographic characteristics, such as gender, age, living conditions and socioeconomic status [5, 6]. Psychiatric conditions and physical illness, as predisposing factors, have also been extensively investigated. Indeed, depression, alcohol abuse, personality and anxiety disorders are the most common psychiatric disorders associated with suicide risk [7] while cancer, stroke, epilepsy and chronic pain conditions are the somatic health conditions commonly associated with suicide-related outcomes [8–12].

Although the wider literature suggests that the majority of those with physical illness has additional psychiatric disorders and those with mental illness are at risk of developing medical/physical health conditions, few suicide-related studies have examined the effect of physical and mental health conditions conjointly [13–15]. Given this gap in knowledge, the aim of the current study was to analyse longitudinal data from Scottish participants and investigate, whether physical/mental health multimorbidity predicts suicidal thoughts and behaviour, and further explore if this effect is stronger than either of the health conditions alone. Specifically, we hypothesised that in comparison to those with no physical or mental health conditions, those with physical/mental multimorbidity would have higher suicide risk (risk of suicidal thoughts and suicide attempts) over the course of the follow-ups.

## Methods

### Setting and participants

**Fig. 1:**
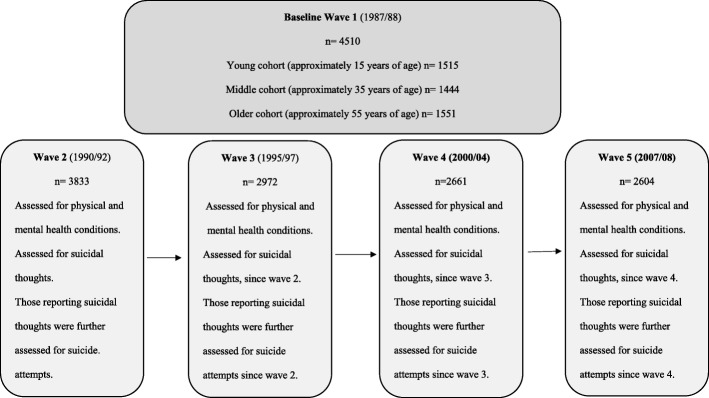
Twenty-07 sample and measurements for baseline and follow-up waves

Secondary analyses of The West of Scotland Twenty-07 Cohort Study (Twenty-07). Twenty-07 was established in 1986 at the Medical Research Council (MRC) Social and Public Health Sciences Unit, University of Glasgow [16]. The aim of the twenty-07 study was to track study participants from the Central Clydeside Conurbation (Glasgow city and environs) across five waves and to investigate the progress of inequalities in health, based on the social factors of gender, age, marital status, social class and area of residence [16]. Twenty-07 involves three cohorts 20 years apart (*n* = 4510) of participants born around 1932, 1952 and 1972. In the baseline study year (1987/88) participants were approximately 15, 35 and 55 years old. As presented in Fig. 1, they were followed up at 1990/92 (Wave 2), at 1995/97 (Wave 3), at 2000/04 (Wave 4) and 2007/08 (Wave 5). Regional and locality samples were included: regional samples were collected by stratifying local districts by census data on socioeconomic groups and unemployment, along with 52 postcode sectors; locality samples were taken from postcode sectors from two areas in Glasgow [16]. For both samples, an enhanced electoral register was used in order to select residents from target age groups [16]. The Twenty-07 study involved self-completion postal questionnaires and structured interviews conducted by nurses trained in the administration of the study protocol; further information on data collection, type of data and collecting process can be found here http://2007study.sphsu.mrc.ac.uk/.

### Measures

**Fig. 2:**
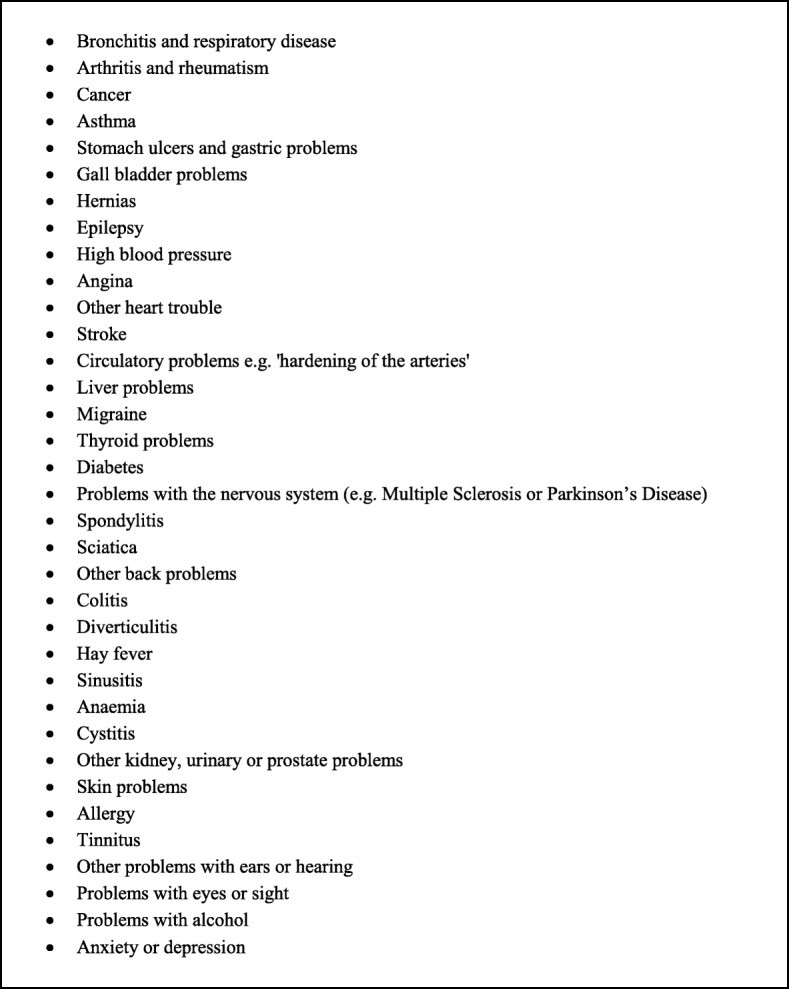
List of common long-standing conditions presented to twenty-07 participants

The presence of long-standing chronic conditions was enquired about during interviews, through a number of open-ended and yes/no questions. Initially participants were asked to report the number of times they visited their doctor or attended an outpatient unit during the past twelve months before the interview. Further, they were specifically asked: “Do you have any long-lasting illness, disability or infirmity? By long-standing I mean anything that has troubled you over a period of time or that is likely to affect you over a period of time?” A more specific question on mental illness was also asked: “Do you suffer from anxiety or depression, or do you have any mental problems, phobias, panics or nervous disorders which you haven’t already told me about?”. As an additional prompt, show cards with common conditions were shown, for which respondents provided a yes/no reply (see Fig. 2). After the information on any long-lasting condition, participants were asked to report their contact with health care services in regard to each health condition mentioned and the limitations they encountered in their day to day activities. Moreover, questions related to prescribed or non-prescribed medication were asked to all twenty-07 participants (regardless of whether they replied positively to the long-standing conditions questions). Those with long-standing conditions were asked to provide further details of each medication or other treatment related to their health conditions reported. All the questions regarding long-standing illness and medication were repeated at each follow-up wave.

Based on a major Scottish study using a large primary care population to explore the level of multimorbidity and its association with socioeconomic deprivation [17], Katikireddi and colleagues further defined the self-reported chronic health conditions of twenty-07 participants [18]. More specifically, two medically qualified authors of the Katikireddi et al. study coded all health conditions based on the coding of the Royal College of General Practitioners’ Morbidity system, along with information on the prescriptions reported by each participant and further verbatim responses [18]. In order to ensure validity and minimise potential attrition bias, the authors modelled their outcomes at each wave and used multiple imputation with chained equations in regard to the missing wave and item data [18]. After data cleaning and coding Katikireddi and colleagues computed variables for all physical and mental health conditions, for each participant at each wave [18]. The derived variables of all health conditions computed by Katikireddi and colleagues were used in our analysis. The proportions of twenty-07 participants with physical and mental health conditions at baseline and at each follow-up wave are given in the online Additional file 1: Table S1.

Questions about suicidal thoughts and behaviours were asked at the first follow-up period (wave 2). Participants were asked: “Have you ever seriously thought about taking an overdose of drugs or injuring yourself deliberately?”. Given the structure of the above question we do not have information on the intent to die as the original twenty-07 question did not enquire about suicidal intent. Indeed, it should be noted that there is no agreed-upon terminology used to describe all aspects of the suicidal and self-harming behaviours; moreover, research indicates those who self-harm – irrespective of whether there is evidence of suicidal intent or not –are at increased risk of future self-harm and suicide, compared to the general population [1, 19–21].

Those replying yes to the suicidal thoughts item were further asked: “Have you ever actually taken an overdose of drugs or injured yourself deliberately?” The above item on previous suicidal behaviours was used in the current study as a suicide attempt question, regardless of the lack of information on the intent to die. In the subsequent follow-up waves (3–5), participants were asked if they had suicidal thoughts and if they had attempted suicide since the last time they were contacted. Specifically, the questions were: “Since we last visited you in (last wave) have you ever seriously thought about taking an overdose of drugs or injuring yourself deliberately?” and “Since we last visited you in (last wave) have you ever actually taken an overdose of drugs or injured yourself deliberately?”. As the follow-up contact was within 5 years since last visit (see Fig. 2), the following additional questions were asked regarding the specific time period of their suicidal thoughts and suicide attempts:

“When was the last time you felt like that?” and “When was the last time that happened?”

As the physical and mental health conditions of participants were considered ‘as current’ at each wave, only suicidal thoughts and suicide attempts within the past year could be used in the present analyses. Therefore, based on the date of interview and the dates given by the participants regarding their own suicidal thoughts and suicide attempts, those who reported suicidal thoughts and attempts within 12 months of their interview were considered as having suicidal thoughts/attempts within the past year. The variables of “past year suicidal thoughts” and “past year suicide attempts” (Yes/No) were computed for each twenty-07 participant and used in the current study. It should be noted that the youngest cohort in the first follow-up wave (wave 2), was not assessed either for suicidal thoughts or attempts and was not included in wave 2 analysis. The items assessing suicidal thoughts and attempts were also combined to yield the derived variable of “past year suicidality”. Although suicidality may not be an appropriate term in clinical settings, due to the low rate of suicide attempts in this sample, to maximise power, we conducted additional analysis which combined suicidal thoughts and suicide attempts into a suicidality variable. However, we note that this approach is not optimal given the increasing evidence highlighting the substantial differences between those who think about suicide and those who attempt suicide [19]. Table 1 presents the number of twenty-07 participants reporting past year suicidal ideation, suicide attempts and suicidality for waves 2 to 5.

**Table 1:** Past year suicidal thoughts, suicide attempts and suicidality in Twenty-07 follow-up waves

	Suicidal thoughts* Yes/No n (%)	Suicide attempts* Yes/No n (%)	Suicidality* Yes/No n (%)
Wave 2	Yes = 41 (1.7%)	Yes = 5 (0.2%)	Yes = 42 (1.7%)
	No = 2427 (98.3%)	No = 2465 (99.8%)	No = 2425 (98.3%)
Wave 3	Yes = 37 (1.7%)	Yes = 9 (0.4%)	Yes = 38 (1.8%)
	No = 2109 (98.3%)	No = 2137 (99.6%)	No = 2107 (98.2%)
Wave 4	Yes = 49 (1.9%)	Yes = 7 (0.3%)	Yes = 49 (1.9%)
	No = 2598 (98.1%)	No = 2647 (99.7%)	No = 2598 (98.1%)
Wave 5	Yes = 56 (2.2%)	Yes = 9 (0.4%)	Yes = 54 (2.1%)
	No = 2498 (97.8%)	No = 2550 (99.6%)	No = 2498 (97.9%)

*Missing replies omitted

In order to explore if suicide risk varies as a function of physical/mental multimorbidity, participants were grouped into four mutually exclusive categories at each wave: those with (1) neither physical nor mental health condition (controls); (2) one or more physical health condition; (3) one or more mental health condition and; (4) multimorbidity. Multimorbidity was defined as the co-occurrence of at least one physical condition and at least one mental health condition. Since the suicidal behaviours items were introduced in wave 2, our analysis of the role of mental/physical multimorbidity in the risk of suicidal thoughts and suicide attempts is restricted to waves 2 to 5.

As previous studies indicate the significant effect of sociodemographic characteristics in the prediction of suicidal behaviour, in a series of logistic regressions we adjusted for sociodemographic characteristics. After re-coding marital status and education levels into the binary variables of “living conditions” (Yes/No) and “higher education” (Yes/No), respectively, the sociodemographic characteristics used in the current study were: age, marital status, education level and economic status. The sociodemographic characteristics of each health condition group and for all waves (1–5), are presented in the online Additional file 1: Table S2.

### Statistical analysis

The original twenty-07 dataset (wide-version) was restructured and its long-version was used for a longitudinal analysis using a generalised estimating equation (GEE) model. In the long version of the dataset repeated measurements over time are represented by separate observations and so tend to be correlated. The GEE model allows for these correlations. Hence, a GEE model, based on the repeated twenty-07 measurements of health conditions and suicide-related items, was used in order to investigate if multimorbidity, physical health conditions only, and mental health conditions only predict suicidal thoughts, suicide attempts and suicidality (either suicide attempts or suicidal ideation). The reference category for the GEE was those with “neither physical nor mental health conditions” and the effect sizes are presented through odds ratios (ORs) and 95% confidence intervals (Cis). Estimated marginal means were utilized to test for differences between multimorbidity and either physical or mental health conditions alone. All the statistical analysis was performed with the Statistical Package for Social Sciences SPSS version 24 (SPSS Inc., Chicago, IL, USA).

### Missing data

As there was some missing data, regarding the dates reported for previous suicidal thoughts and behaviours, the computed items of “past year suicidal thoughts” and “past year suicide attempts”, were expanded into three versions and sensitivity analyses were conducted. More specifically, in the main version (Table 1), when the dates of last having either suicidal thoughts or attempted suicide were missing, the derived variables of “past year suicidal thoughts” and “past year suicide attempts” did not include the cases with those missing replies. Within the sensitivity analysis and in the second version, all missing replies were treated as a past year suicidal behaviour (within 12 months of the interview). For the third version, missing replies were treated as taking place more than a year ago (more than 12 months since the interview). These sensitivity analyses based on the second and third modified versions are contained in the online Additional file 1: Table S3 and Table S4.

A small number of participants who took part in the twenty-07 waves of data collection had missing data on the occurrence of suicidal thoughts (4 participants in wave 2; 1 participant in wave 3; 7 participants in wave 4; and 8 participants in wave 5). In order to test the differences of those with these missing data, compared to those who responded, a further GEE analysis was conducted with a binary outcome indicating those missing, and adjusted for all health groups and the sociodemographics of age and social class. Our results indicated that the missing replies were related to the health groups of multimorbidity and mental health only (multimorbidity: OR: 11.109, 95% CI 2.249–54.878, *p* = 0.003; mental health conditions only: OR: 18.299, 95% CI 3.369–99.394, *p* = 0.001) but not to age or social class. Hence, in our analyses these cases were missing at random (MAR).

## Results

### Demographic characteristics, frequency of suicidal thoughts and suicide attempts of participants with multimorbidity across waves

As presented in the online Additional file 1: Table S2, half of the multimorbidity group in each wave were female and most of the participants in this group were married. While in waves 2 and 3 a considerable proportion of those with multimorbidity were employed (34 and 37.5% respectively), at waves 4 and 5 around 40% of those with multimorbidity were not in the labour force, partly due to members of the older cohorts being retired by the time of the last follow-up periods. In terms of education level, the highest level of education for those with multimorbidity issues was achieved by waves 3 and 4.

The largest proportion of participants having thoughts about suicide was among those identified as multimorbid. More specifically, people with multimorbidity accounted for 53.7% of those reporting suicidal thoughts at wave 2 (*n* = 41), 62.2% of those having past year suicidal thoughts at wave 3 (*n* = 37), 63.3% of those in wave 4 (*n* = 49) and 66.1% of those in wave 5 (*n* = 56). Those with only mental health conditions accounted for 14.6% of the total number of people having suicidal thoughts at wave 2, 16.2% of those having past year suicidal thoughts at wave 3, 16.3% of those in wave 4 and 21.4% of those in wave 5. Those with only physical health conditions accounted for 22% of those with past year thoughts of suicide at wave 2, 5.4% of those in wave 3, 14.3% of those in wave 4 and 10.7% of those in wave 5.

For past year suicide attempts overall, the number of cases was low, with 5 people reporting suicide attempts at wave 2 (multimorbidity *n* = 2; mental health condition only n = 2, physical health condition only *n* = 1), 9 participants at wave 3 (multimorbidity *n* = 3; mental health condition only n = 2, physical health condition only n = 1; neither physical nor mental n = 3), 7 participants at wave 4 (multimorbidity *n* = 4; mental health condition only n = 2, neither physical nor mental n = 1) and 9 participants at wave 5 (multimorbidity *n* = 6; mental health condition only *n* = 3).

### Generalised estimating equation analyses of the predictive role of physical/mental multimorbidity in the risk of suicidality

Three separate generalised estimating equation (GEE) models were used in order to investigate the role of health groupings (multimorbidity, physical health conditions only, mental health conditions only) in the prediction of suicidal thoughts, suicide attempts and suicidality (Table 2). The GEE analysis included the health groupings and suicide-related items reported for waves 2 to 5. Multimorbidity had a significant association with all the suicide risk indices.

**Table 2:** Generalised estimating equation model on the predictive role of multimorbidity, physical and mental health conditions in the risk of suicide-related outcomes among twenty-07 participants

	Suicidal thoughts			Suicide attempts			Suicidality		
	OR	95% CI	*p*	OR	95% CI	*p*	OR	95% CI	*p*
Neither physical nor mental conditions	1 (ref)	–	–	1 (ref)	–	–	1 (ref)	–	–
Physical conditions only	0.987	0.534–1.826	0.967	0.309	0.058–1.644	0.169	1.030	0.559–1.898	0.923
Mental conditions only	17.482	9.324–32.778	< 0.001	17.593	4.805–64.409	< 0.001	17.953	9.612–33.534	< 0.001
Multimorbidity ^a^	16.407	9.574–28.116	< 0.001	8.279	2.735–25.060	< 0.001	16.103	9.392–27.611	< 0.001

^a^One or more mental and one or more physical illness in the same person

While having physical health conditions was not associated with any suicide-related item (Table 2), having a mental health condition was a significant predictor of suicidal thoughts, suicide attempts and suicidality, compared to having neither physical nor mental health conditions.

### Adjusted generalised estimating equation analyses of the predictive role of multimorbidity in suicidality

After adjustment for the sociodemographic characteristics of sex, age, living conditions, education level, employment status and social class, multimorbidity remained significantly associated with all suicide-related items (Table 3). Although having only mental health conditions was additionally associated with suicidal thoughts, suicide attempts and suicidality, the role of physical health conditions was not found to be predictive of either suicide-related item (Table 3).

**Table 3:** Adjusted generalised estimating equation model on the predictive role of multimorbidity, physical and mental health conditions in the risk of suicidality among twenty-07 participants

	Suicidal thoughts^1^			Suicide attempts^1^			Suicidality^1^		
	OR	95% CI	*p*	OR	95% CI	*p*	OR	95% CI	*p*
Neither physical nor mental conditions	1 (ref)	–	–	1 (ref)	–	–	1 (ref)	–	–
Physical conditions only	1.172	0.551–2.497	0.680	0.364	0.039–3.417	0.377	1.264	0.599–2.668	0.538
Mental conditions only	15.268	7.389–31.550	< 0.001	14.187	3.671–54.830	< 0.001	16.098	7.844–33.039	< 0.001
Multimorbidity^a^	17.209	8.814–33.603	< 0.001	5.776	1.427–23.383	0.014	17.135	8.772–33.473	< 0.001

^1^Adjusted for sociodemographic characteristics: sex, age, living conditions, any standard/high educational qualification, employment status, current social class

^a^One or more mental and one or more physical illness in the same person

### The effect of physical/mental health multimorbidity in suicidal thoughts and suicide attempts

In order to test whether the effect of physical/mental multimorbidity on suicide risk is stronger than either of health conditions alone, we performed pairwise comparisons between multimorbidity, mental health conditions only, physical health conditions only and controls, using estimated marginal means. Our analyses on the 20 year follow-up period of twenty-07, indicated that multimorbidity does not increase the risk of suicidal ideation or suicide attempts, more than mental illness alone (suicidal thoughts *p* = 0.776; suicide attempts *p* = 0.136).

### Sensitivity analysis

A number of generalised estimating equation (GEE) models investigating the role of health groupings in the prediction of suicidal thoughts and behaviours were conducted, based on the two modified versions of missing data. The results of the unadjusted and adjusted GEE analyses, for both versions, were in accordance with the findings based on the main version where multimorbidity was found to be significantly associated with all suicide-related indices. Specifically, for the second modified version where missing values were treated as a past year thought and behaviour, multimorbidity and mental health had a significant association with all the suicide-related risk indices, whereas physical health conditions were not significantly associated with any suicide-related outcome (Additional file 1: Tables S5-S6). For the third version, where missing replies were treated as taking place more than a year before the interview, the findings were consistent, only the multimorbidity and mental health categories were significantly associated with suicidal thoughts and suicide attempts (Additional file 1: Tables S7-S8).

## Discussion

The West of Scotland Twenty-07 cohort study was analysed, and our results indicated that having co-occurring physical and mental health conditions increases the risk of suicidality; specifically, our findings indicated that physical/mental multimorbidity could be potentially used as a predictor of suicidal thoughts and behaviours. Due to the small number of cases of suicidal behaviour, the current findings about the role of multimorbidity in the prediction of suicide attempts should be interpreted with caution. Moreover, multimorbidity did not have a stronger effect than mental health conditions alone; interestingly, physical health conditions were not associated with any suicide-related item.

The relationship between suicide risk and multimorbidity, characterised by the co-presence of mental and physical illness, has not been extensively investigated. One of the few previous studies on the risk of suicide among populations with health conditions indicated that the co-existence of a mental and a physical illness diagnosis, regardless of which one is given first, is a significant risk factor for suicide [22]. These authors reported that the temporality of conditions is additionally important in clinical settings, as a psychiatric diagnosis coming years after a somatic condition provides higher risk of suicide, than having a somatic condition developed after a psychiatric diagnosis. Although our results are in accordance with the finding that the risk of suicidal thinking and behaviour is increased due to physical/mental multimorbidity, it was not feasible to investigate the onset of health conditions in our twenty-07 dataset and further exploration of the timing of condition onset is required.

While previous multimorbidity research mainly focused on the risk of suicide attempts and deaths by suicide [22, 23] our findings shed more light on the risk of suicidal thoughts (rather than suicide attempts) for those with multimorbidity issues and confirm previous findings. Based on previous research, a US (United States) study indicated that when pulmonary disease co-occurs with depression, the risk of suicidal thoughts is higher than having neither of the under-studied health conditions [24]; however, the same study further indicated that the risk of suicidal thoughts for pulmonary disease and depression co-occurrence is higher, compared to having either pulmonary disease or depression alone.

In terms of the association of physical/mental health co-occurrence and the risk of suicide attempts, Fuller-Thomson and colleagues highlighted that within the context of other factors (childhood adversities, current pain), those with arthritis, lifetime depression, anxiety or substance dependency have higher odds of suicide attempts, compared to those without arthritis [25]. However, as noted earlier, the small numbers of suicide attempts in twenty-07 sample limited the power of our analyses and we cannot draw any concrete conclusions about the association between the predictors–outcomes of interest.

Overall, our findings on the risk of suicidal thoughts and behaviour in multimorbid populations could be potentially related to the burden of multiple diseases that affect both physical and mental health. Although the co-existence of physical and mental illness is relatively prevalent, and it has an effect on mortality outcomes [13, 26–29] it is not yet clear how the co-occurrence of these health conditions influences suicidal thinking and related behaviour. Further studies investigating feelings of entrapment in a stressful situation related to coping with multiple physical and mental health conditions, would help us understand the progress of suicidal thoughts in these vulnerable populations [1, 30]. Even though our findings on the risk of suicide attempts are weak due to the small numbers, further exploration is required to investigate whether multimorbidity predicts the transition from thoughts to attempts and if having access to multiple medications, is a factor influencing the development of suicidal plans and behaviours.

Considering whether physical/mental illness co-occurrence increases the risk of suicidal behaviours more than either condition alone, our longitudinal analyses highlighted that multimorbidity is not a stronger predictor of suicidal ideation or suicide attempts than mental illness alone. Taken together and consistent with our earlier cross-sectional work [31] our findings suggest that physical and mental health conditions do not work additively to increase the risk of suicidal ideation or attempts. As previous studies have highlighted that the temporality of health conditions differentiates suicide risk [22] further prospective studies investigating the risk of either suicidal thoughts, suicide attempts or suicide among multimorbid groups, should address the onset of physical and mental health conditions.

Considering the multimorbidity literature indicates that the prevalence of multiple health conditions increases with age [17], multimorbidity effects on suicide-related outcomes are likely to be stronger in older versus younger populations. However, there is a considerable research gap on whether age differentiates the effect of multimorbidity on suicidal thoughts and behaviours, Given the limited number of suicidality cases in our study, in a larger sample, future research should explore whether the effect of multimorbidity on suicidal thoughts and suicide attempts in moderated by age. Multimorbidity effects are likely to be stronger in older versus middle-aged and younger populations.

One of the major strengths of the current study is the use of longitudinal data that can advance our knowledge of the relationship between physical/mental health conditions and suicidality, over time. Considering the lack of pertinent longitudinal studies in general population samples of people experiencing physical/mental illness co-occurrence, the twenty-07 cohort study provides a robust longitudinal test of the association. The results from the twenty-07 cohort should be viewed in light of potential limitations. As this study employed secondary analysis attention should be given to the fact that twenty-07 was not designed to address the specific research questions addressed here. For the coding of all the physical and mental health conditions, medication information and verbatim responses were used by trained health professionals. However, it should be noted that the initial use of the self-reported health information may limit the generalisability of our findings, in terms of the severity level of each condition. Future studies should investigate whether the severity of different physical and mental illnesses differentiates the risk of suicidal thoughts and behaviours.

In addition, given the self-reported nature of all health conditions, we cannot rule out that either the physical or the mental health conditions were over- or under-reported, and this may have influenced our findings. Furthermore, members of the reference group with neither physical nor mental health conditions could potentially have had other health issues that were not reported or assessed during the data collection. Although widely used, an additional limitation that should be highlighted is the assessment of past suicidal thoughts and suicide attempts, which was based on single questions rather than a suicide risk assessment scale. Although there is no consistent terminology for suicide-related outcomes, the current study was limited by the fact that the items used in the twenty-07 study, lack of information on intent to die. Also, as noted earlier, the number of suicide attempt cases was small, consequently no firm conclusions could be made about the role of multimorbidity in the risk of suicide attempts. This latter limitation underlines the importance of further replication with larger datasets.

## Conclusions

While suicide prevention strategies have been developed for different populations at risk of suicide, no clear evidence exists for the risk of multimorbid patients. In the present study of a sample representative of the Scottish population, we found that physical/mental multimorbidity is a risk factor for suicidal thoughts and suicide attempts but not beyond the effects of mental illness. Our results potentially suggest that health professionals in primary and secondary care should screen their patients with physical and mental illness co-occurrence for suicidal thoughts and behaviours. Considering the prevalence of physical/mental multimorbidity in primary care, further targeting and intervention is required.

## Additional file

Additional file 1: **Table S1.** Proportion of twenty-07 participants with current physical and mental health conditions at each wave. **Table S2.** Sociodemographic characteristics of twenty-07 participants with physical only, mental only, multimorbidity and neither physical nor mental health conditions, at follow-up waves 2–5. **Table S3.** Sensitivity analyses of past year suicidal thoughts, suicide attempts and suicidality for twenty-07 follow-up waves (version 2). **Table S4.** Sensitivity analyses of past year suicidal thoughts, suicide attempts and suicidality for twenty-07 follow-up waves (version 3). **Table S5.** Generalised estimating equation model on the predictive role of multimorbidity, physical and mental health conditions in the risk of suicide-related outcomes among twenty-07 participants (version 2). **Table S6.** Adjusted generalised estimating equation model on the predictive role of multimorbidity, physical and mental health conditions in the risk of suicidality among twenty-07 participants (version 2). **Table S7.** Generalised estimating equation model on the predictive role of multimorbidity, physical and mental health conditions in the risk of suicide-related outcomes among twenty-07 participants (version 3). **Table S8.** Adjusted generalised estimating equation model on the predictive role of multimorbidity, physical and mental health conditions in the risk of suicidality among twenty-07 participants (version 3). (DOCX 52 kb)

## Abbreviations

95% CI: 95% Confidence Interval
GEE: Generalised Estimating Equation model
MAR: Missing At Random
MRC: Medical Research Council
OR: Odds Ratio
Twenty-07: The West of Scotland Twenty-07 Cohort Study
US: United States
