# Supplementary material for: The role of physical and mental multimorbidity in suicidal thoughts and behaviours in a Scottish population cohort study
Source: BMC Psychiatry. 2019 Jan 23;19:38. doi: 10.1186/s12888-019-2032-8 (PMC6344985; doi:10.1186/s12888-019-2032-8)
Supplement: Supplementary file 1 — Table S1. Proportion of twenty-07 participants with current physical and mental health conditions at each wave. Table S2. Sociodemographic characteristics of twenty-07 participants with physical only, mental only, multimorbidity and neither physical nor mental health conditions, at follow-up waves 2–5. Table S3. Sensitivity analyses of past year suicidal thoughts, suicide attempts and suicidality for twenty-07 follow-up waves (version 2). Table S4. Sensitivity analyses of past year suicidal thoughts, suicide attempts and suicidality for twenty-07 follow-up waves (version 3). Table S5. Generalised estimating equation model on the predictive role of multimorbidity, physical and mental health conditions in the risk of suicide-related outcomes among twenty-07 participants (version 2). Table S6. Adjusted generalised estimating equation model on the predictive role of multimorbidity, physical and mental health conditions in the risk of suicidality among twenty-07 participants (version 2). Table S7. Generalised estimating equation model on the predictive role of multimorbidity, physical and mental health conditions in the risk of suicide-related outcomes among twenty-07 participants (version 3). Table S8. Adjusted generalised estimating equation model on the predictive role of multimorbidity, physical and mental health conditions in the risk of suicidality among twenty-07 participants (version 3). (DOCX 52 kb) [file 12888_2019_2032_MOESM1_ESM.docx]

Additional file 1

Table S1. Proportion of twenty-07 participants with current physical and mental health conditions at each wave

| **Conditions** | **Baseline**  **(n=4510)**  **n (%)** | **Wave 2**  **(n=3833)**  **n (%)** | **Wave 3**  **(n=2972)**  **n (%)** | **Wave 4**  **(n=2661)**  **n (%)** | **Wave 5 (n=2604)**  **n (%)** |
| --- | --- | --- | --- | --- | --- |
| **Mental health conditions** | | | | | |
| Alcohol | 14 (0.3) | 26 (0.7) | 25 (0.8) | 40 (1.5) | 40 (1.5) |
| Drunkenness | 1 (0) | 2 (0.1) | - | 1 (0) | 1 (0) |
| Anorexia | 2 (0) | 3 (0.1) | 3 (0.1) | 3 (0.1) | 2 (0.1) |
| Anxiety | 152 (3.4) | 226 (5.9) | 201 (6.8) | 199 (7.5) | 310 (11.9) |
| Dementia | 2 (0) | - | - | - | 7 (0.3) |
| Depression | 235 (5.2) | 278 (7.3) | 185 (6.2) | 240 (9) | 339 (13) |
| Learning disability | 7 (0.2) | 7 (0.2) | 6 (0.2) | 5 (0.2) | 6 (0.2) |
| Schizophrenia | 9 (0.2) | 10 (0.3) | 11 (0.4) | 15 (0.6) | 24 (0.9) |
| Substance abuse | 3 (0.1) | 3 (0.1) | 5 (0.2) | 5 (0.2) | 7 (0.3) |
| **Physical health conditions** | | | | | |
| Asthma | 160 (3.5) | 158 (4.1) | 156 (5.2) | 181 (6.8) | 190 (7.3) |
| Atrial | 2 (0) | 2 (0.1) | 4 (0.1) | 11 (0.4) | 19 (0.7) |
| Blind | 17 (0.4) | 21 (0.5) | 23 (0.8) | 24 (0.9) | 38 (1.5) |
| Bronchitis | 3 (0.1) | 2 (0.1) | 2 (0.1) | 3 (0.1) | 3 (0.1) |
| Cancer | 29 (0.6) | 37 (1) | 44 (1.5) | 58 (2.2) | 93 (3.6) |
| Coronary Heart Disease | 141 (3.1) | 171 (4.5) | 172 (5.8) | 181 (6.8) | 213 (8.2) |
| Constipation | 24 (0.5) | 40 (1) | 12 (0.4) | 2 (0.1) | 4 (0.2) |
| Chronic Obstructive Pulmonary Disease | 48 (1.1) | 49 (1.3) | 45 (1.5) | 40 (1.5) | 66 (2.5) |
| Diabetes | 41 (0.9) | 48 (1.3) | 64 (2.2) | 99 (3.7) | 156 (6) |
| Dyspepsia | 200 (4.4) | 191 (5) | 130 (4.4) | 135 (5.1) | 145 (5.6) |
| Epilepsy | 37 (0.8) | 39 (!) | 32 (1.1) | 29 (1.1) | 27 (1) |
| Glaucoma | 11 (0.2) | 13 (0.3) | 18 (0.6) | 22 (0.8) | 41 (1.6) |
| Heart failure | 11 (0.2) | 12 (0.3) | 10 (0.3) | 13 (0.5) | 25 (1) |
| Hepatitis | 1 (0) | - | 1 (0) | 2 (0.1) | - |
| Hypertension | 228 (5.1) | 295 (7.7) | 299 (10.1) | 448 (16.8) | 641 (24.6) |
| Inflammatory Bowel Disease (IBD) | 20 (0.4) | 22 (0.6) | 23 (0.8) | 27 (1) | 31 (1.2) |
| Irritable Bowel Syndrome | 19 (0.4) | 39 (1) | 59 (2) | 64 (2.4) | 73 (2.8) |
| Intestine | 18 (0.4) | 27 (0.7) | 42 (1.4) | 61 (2.3) | 90 (3.5) |
| Kidney | 3 (0.1) | 4 (0.1) | 7 (0.2) | 4 (0.2) | 17 (0.7) |
| Liver | 3 (0.1) | 4 (0.1) | 2 (0.1) | 6 (0.2) | 11 (0.4) |
| Migraine | 191 (4.2) | 201 (5.2) | 161 (5.4) | 193 (7.3) | 201 (7.7) |
| Multiple sclerosis | 8 (0.2) | 9 (0.2) | 12 (0.4) | 10 (0.4) | 15 (0.6) |
| Parkinson | 3 (0.1) | 2 (0.1) | 1 (0) | 2 (0.1) | 21 (0.8) |
| Prostate | 13 (0.3) | 28 (0.7) | 34 (1.1) | 42 (1.6) | 52 (2) |
| Psoriasis | 127 (2.8) | 169 (4.4) | 149 (5) | 216 (8.1) | 226 (8.7) |
| Sinusitis | 7 (0.2) | 11 (0.3) | 16 (0.5) | 9 (0.3) | 16 (0.6) |
| Stroke | 21 (0.5) | 23 (0.6) | 39 (1.3) | 52 (2) | 80 (3.1) |
| Thyroid | 46 (1) | 58 (1.5) | 65 (2.2) | 93 (3.5) | 154 (5.9) |
| Vascular disease | 30 (0.7) | 46 (1.2) | 55 (1.9) | 78 (2.9) | 103 (4) |
| Hearing problems | 72 (1.6) | 184 (4.8) | 193 (6.5) | 236 (8.9) | 284 (10.9) |
| Inflammatory problems | 40 (0.9) | 86 (2.2) | 66 (2.2) | 88 (3.3) | 116 (4.5) |
| Pain | 700 (15.5) | 821 (21.6) | 785 (26.6) | 777 (29.3) | 875 (33.6) |

Table S2. Sociodemographic characteristics of twenty-07 participants with physical only, mental only, multimorbidity and neither physical nor mental health conditions, at follow-up waves 2-5

|  | **Physical health conditions only** | **Mental health conditions only** | **Multimorbidity** | **Neither physical nor mental health conditions** |
| --- | --- | --- | --- | --- |
| **Characteristics*** | **Wave 2** | | | |
| **Age** **M (SD)** | 49.6 (12.8) | 30.8 (14.6) | 50 (12.5) | 30.4 (14.9) |
| **Sex n (%)**  Male  Female | 643 (45.6%)  768 (54.4%) | 66 (33.5%)  131 (66.5%) | 125 (39.7%)  190 (60.3%) | 932 (48.8%)  978 (51.2%) |
| **Marital status n (%)**  Never married  Married  Divorced/separated  Widowed | 206 (14.6%)  996 (70.7%)  113 (8%)  93 (6.6%) | 119 (60.4%)  53 (26.9%)  21 (10.7%)  4 (2%) | 56 (17.8%)  178 (56.5%)  43 (13.7%)  38 (12.1%) | 1172 (61.4%)  625 (32.8%)  78 (4.1%)  33 (1.7%) |
| **Education level achieved**^[[1]](#footnote-1)^ **n (%)**  None  Standard education or equivalent  Higher education | 12 (10.6%)  101 (89.4%)  - | 11 (10.2%)  97 (89.8%)  - | 3 (13%)  20 (87%)  - | 86 (7.8%)  1011 (92%)  2 (0.2%) |
| **Economic status n (%)**  Not in the labour force  Employed  Unemployed  Not working on account of illness/disability | 388 (27.9%)  757 (54.5%)  90 (6.5%)  155 (11.2%) | 62 (31.8%)  91 (46.7%)  31 (15.9%)  11 (5.6%) | 90 (29.5%)  103 (33.8%)  37 (12.1%)  75 (24.6%) | 534 (28.1%)  1156 (60.8%)  201 (10.6%)  9 (0.5%) |
|  | **Wave 3** | | | |
| **Age** **M (SD)** | 54.8 (12.1) | 38.5 (14.6) | 55.7 (11.7) | 37.5 (14.5) |
| **Sex n (%)**  Male  Female | 582 (43.9%)  744 (56.1%) | 39 (37.9%)  64 (62.1%) | 98 (34.9%)  183 (65.1%) | 606 (48%0  656 (52%) |
| **Marital status n (%)**  Never married  Married  Divorced/separated  Widowed | 213 (20.9%)  649 (63.8%)  75 (7.4%)  80 (7.9%) | 32 (36.8%)  39 (44.8%)  14 (16.1%)  2 (2.3%) | 57 (21.6%)  142 (53.8%)  29 (11%)  36 (13.6%) | 362 (46.4%)  357 (45.7%)  39 (5%)  23 (2.9%) |
| **Education level achieved n (%)**  None  Standard education or equivalent  Higher education | -  160 (46%)  188 (54%) | -  23 (51.1%)  22 (48.9%) | -  36 (48.6%)  38 (51.4%) | -  195 (41.1%)  280 (58.9%) |
| **Economic status n (%)**  Not in the labour force  Employed  Unemployed  Not working on account of illness/disability | 521 (40.5%)  595 (46.3%)  58 (4.5%)  111 (8.6%) | 19 (19.4%)  57 (58.2%)  11 (11.2%)  11 (11.2%) | 87 (32.2%)  101 (37.4%)  19 (7%)  63 (23.3%) | 256 (20.7%)  915 (74%)  49 (4%)  17 (1.4%) |
|  | **Wave 4** | | | |
| **Age M (SD)** | 59.1 (11.9) | 41.9 (14.1) | 58.5 (11.7) | 42.3 (14.1) |
| **Sex n (%)**  Male  Female | 618 (45%)  755 (55%) | 41 (39%)  64 (61%) | 119 (34.8%)  223 (65.2%) | 420 (49.9%)  421 (50.1%) |
| **Marital status n (%)**  Never married  Married  Divorced/separated  Widowed | 209 (15.3%)  896 (65.7%)  102 (7.5%)  157 (11.5%) | 40 (38.1%)  54 (51.4%)  7 (6.7%)  4 (3.8%) | 69 (20.4%)  172 (50.7%)  54 (15.9%)  44 (13%) | 273 (32.9%)  480 (57.9%)  52 (6.3%)  24 (2.9%) |
| **Education level achieved n (%)**  None  Standard education or equivalent  Higher education | 64 (26%)  62 (25.2%)  120 (48.8%) | 20 (33.9%)  21 (35.6%)  18 (30.5%) | 21 (26.9%)  23 (29.5%)  34 (43.6%) | 81 (20.4%)  105 (26.4%)  212 (53.3%) |
| **Economic status n (%)**  Not in the labour force  Employed  Unemployed  Not working on account of illness/disability | 619 (45.5%)  668 (49.2%)  31 (2.3%)  41 (3%) | 14 (13.7%)  73 (71.6%)  4 (3.9%)  11 (10.8%) | 136 (40.5%)  136 (40.5%)  14 (4.2%)  50 (14.9%) | 110 (13.3%)  680 (82.3%)  30 (3.6%)  6 (0.7%) |
|  | **Wave 5** | | | |
| **Age M (SD)** | 64.2 (12.7) | 48 (13.5) | 63.4 (12.5) | 47.9 (13.7) |
| **Sex n (%)**  Male  Female | 596 (43.9%)  762 (56.1%) | 44 (40.4%)  65 (59.6%) | 183 (37.7%)  302 (62.3%) | 337 (51.7%)  315 (48.3%) |
| **Marital status n (%)**  Never married  Married  Divorced/separated  Widowed | 174 (12.8%)  859 (63.3%)  131 (9.7%)  192 (14.2%) | 34 (31.5%)  52 (48.1%)  19 (17.6%)  3 (2.8%) | 107 (22.1%)  219 (45.2%)  80 (16.5%)  78 (16.1%) | 139 (21.5%)  422 (65.3%)  61 (9.4%)  24 (3.7%) |
| **Education level achieved n (%)**  None  Standard education or equivalent  Higher education | 277 (21.7%)  539 (42.3%)  459 (36%) | 17 (16%)  60 (56.6%)  29 (27.4%) | 152 (33.5%)  164 (36.1%)  138 (30.4%) | 61 (10%)  226 (37.1%)  322 (52.9%) |
| **Economic status n (%)**  Not in the labour force  Employed  Unemployed  Not working on account of illness/disability | 559 (42%)  711 (53.4%)  18 (1.4%)  43 (3.2%) | 12 (12%)  68 (68%)  12 (12%)  8 (8%) | 186 (39.7%)  177 (37.7%)  20 (4.3%)  86 (18.3%) | 70 (11.2%)  544 (86.9%)  9 (1.4%)  3 (0.5%) |

* percent (%) is based on the omission of missing values

Table S3. Sensitivity analyses of past year suicidal thoughts, suicide attempts and suicidality for twenty-07 follow-up waves (version 2).

|  | Suicidal thoughts* | Suicide attempts | Suicidality |
| --- | --- | --- | --- |
| Wave b | Yes = 45 (1.8%)  No = 2427 (98.2%) | Yes = 6 (0.2%)  No =2466 (99.8%) | Yes = 47 (1.9%)  No = 2425 (98.1%) |
| Wave c | Yes = 38 (1.8%)  No = 2109 (98.2%) | Yes = 10 (0.5%)  No = 2137 (99.5%) | Yes = 39 (1.8%)  No = 2107 (98.2%) |
| Wave d | Yes = 56 (2.1%)  No = 2598 (97.9%) | Yes = 7 (0.3%)  No = 2647 (99.7%) | Yes = 56 (2.1%)  No = 2598 (97.9%) |
| Wave e | Yes = 64 (2.5%)  No = 2498 (97.5%) | Yes = 12 (0.5%)  No = 2550 (99.5%) | Yes = 64 (2.5%)  No = 2498 (97.5%) |

* Missing replies treated as taking place the past year (within the last 12 months).

Table S4. Sensitivity analyses of past year suicidal thoughts, suicide attempts and suicidality for twenty-07 follow-up waves (version 3).

|  | Suicidal thoughts* | Suicide attempts | Suicidality |
| --- | --- | --- | --- |
| Wave b | Yes = 41 (1.7%)  No =2431 (98.3%) | Yes = 5 (0.2%)  No = 2467 (99.8%) | Yes = 42 (1.7%)  No = 2430 (98.3%) |
| Wave c | Yes = 37 (1.7%)  No = 2110 (98.3%) | Yes = 9 (0.4%)  No = 2138 (99.6%) | Yes = 38 (1.8%)  No = 2108 (98.2%) |
| Wave d | Yes = 49 (1.8%)  No = 2605 (98.2%) | Yes = 7 (0.3%)  No = 2647 (99.7%) | Yes = 49 (1.8%)  No = 2605 (98.2%) |
| Wave e | Yes = 56 (2.2%)  No = 2506 (97.8%) | Yes = 9 (0.4%)  No = 2553 (99.6%) | Yes = 56 (2.2%)  No = 2506 (97.8%) |

*Missing replies treated as taking place more than a year ago (not within the last 12 months).

Table S5. Generalised estimating equation model on the predictive role of multimorbidity, physical and mental health conditions in the risk of suicide-related outcomes among twenty-07 participants (version 2).

|  | Suicidal thoughts | |  | Suicide attempts | |  | Suicidality | |  |
| --- | --- | --- | --- | --- | --- | --- | --- | --- | --- |
|  | **OR** | **95% CI** | ***p*** | **OR** | **95% CI** | ***p*** | **OR** | **95% CI** | ***p*** |
| Neither physical nor mental conditions | 1 (ref) | - | - | 1 (ref) | - | - | 1 (ref) | - | - |
| Physical conditions only | 0.965 | 0.548-1.701 | 0.903 | 0.463 | 0.106-2.021 | 0.305 | 1.038 | 0.593-1.816 | 0.896 |
| Mental conditions only | 16.227 | 8.940-29.453 | <0.001 | 17.590 | 4.814-64.274 | <0.001 | 16.681 | 9.230-30.148 | <0.001 |
| Multimorbidity ^a^ | 15.060 | 9.174-24.722 | <0.001 | 10.508 | 3.568-30.945 | <0.001 | 15.104 | 9.204-24.786 | <0.001 |

^a^One or more mental and one or more physical illness in the same person

Table S6. Adjusted generalised estimating equation model on the predictive role of multimorbidity, physical and mental health conditions in the risk of suicidality among twenty-07 participants (version 2).

|  | Suicidal thoughts^1^ | |  | Suicide attempts^1^ | |  | Suicidality^1^ | |  |
| --- | --- | --- | --- | --- | --- | --- | --- | --- | --- |
|  | **OR** | **95% CI** | ***p*** | **OR** | **95% CI** | ***p*** | **OR** | **95% CI** | ***p*** |
| Neither physical nor mental conditions | 1 (ref) | - | - | 1 (ref) | - | - | 1 (ref) | - | - |
| Physical conditions only | 1.152 | 0.572-2.322 | 0.692 | 0.342 | 0.036-3.198 | 0.347 | 1.234 | 0.617-2.471 | 0.552 |
| Mental conditions only | 15.767 | 7.963-31.220 | <0.001 | 13.475 | 3.535-51.366 | <0.001 | 16.490 | 8.375-2.469 | <0.001 |
| Multimorbidity ^a^ | 16.203 | 8.679-30.249 | <0.001 | 7.684 | 2.222-26.566 | 0.001 | 16.407 | 8.797-30.602 | <0.001 |

^1^ Adjusted for sociodemographic characteristics: sex, age, living conditions, any standard/high educational qualification, employment status, current social class

^a^One or more mental and one or more physical illness in the same person

Table S7. Generalised estimating equation model on the predictive role of multimorbidity, physical and mental health conditions in the risk of suicide-related outcomes among twenty-07 participants (version 3).

|  | Suicidal thoughts | |  | Suicide attempts | |  | Suicidality | |  |
| --- | --- | --- | --- | --- | --- | --- | --- | --- | --- |
|  | **OR** | **95% CI** | ***p*** | **OR** | **95% CI** | ***p*** | **OR** | **95% CI** | ***p*** |
| Neither physical nor mental conditions | 1 (ref) | - | - | 1 (ref) | - | - | 1 (ref) | - | - |
| Physical conditions only | 0.965 | 0.548-1.701 | 0.903 | 0.463 | 0.106-2.021 | 0.305 | 1.038 | 0.593-1.816 | 0.896 |
| Mental conditions only | 16.227 | 8.940-29.453 | <0.001 | 17.590 | 4.814-64.274 | <0.001 | 16.681 | 9.230-30.148 | <0.001 |
| Multimorbidity ^a^ | 15.060 | 9.174-24.722 | <0.001 | 10.508 | 3.568-30.945 | <0.001 | 15.104 | 9.204-24.786 | <0.001 |

^a^One or more mental and one or more physical illness in the same person

Table S8. Adjusted generalised estimating equation model on the predictive role of multimorbidity, physical and mental health conditions in the risk of suicidality among twenty-07 participants (version 3).

|  | Suicidal thoughts^1^ | |  | Suicide attempts^1^ | |  | Suicidality^1^ | |  |
| --- | --- | --- | --- | --- | --- | --- | --- | --- | --- |
|  | **OR** | **95% CI** | ***p*** | **OR** | **95% CI** | ***p*** | **OR** | **95% CI** | ***p*** |
| Neither physical nor mental conditions | 1 (ref) | - | - | 1 (ref) | - | - | 1 (ref) | - | - |
| Physical conditions only | 0.967 | 0.533-1.826 | 0.967 | 0.309 | 0.058-1.643 | 0.168 | 1.031 | 0.560-1.899 | 0.922 |
| Mental conditions only | 17.281 | 9.219-32.392 | <0.001 | 17.593 | 4.805-64.410 | <0.001 | 17.778 | 9.521-33.194 | <0.001 |
| Multimorbidity ^a^ | 16.303 | 9.516-27.933 | <0.001 | 8.254 | 2.727-24.986 | 0.001 | 16.332 | 9.535-27.974 | <0.001 |

^1^ Adjusted for sociodemographic characteristics: sex, age, living conditions, any standard/high educational qualification, employment status, current social class

^a^One or more mental and one or more physical illness in the same person

1. The item related to the level of education achieved at each follow-up, refers to the period from last wave until the current one questioned [↑](#footnote-ref-1)
